# A quantitative assessment of the consistency of projections from five mathematical models of the HIV epidemic in South Africa: a model comparison study

**DOI:** 10.1186/s12889-023-16995-9

**Published:** 2023-10-27

**Authors:** Haroon Moolla, Andrew Phillips, Debra ten Brink, Edinah Mudimu, John Stover, Loveleen Bansi-Matharu, Rowan Martin-Hughes, Nisaa Wulan, Valentina Cambiano, Jennifer Smith, Anna Bershteyn, Gesine Meyer-Rath, Lise Jamieson, Leigh F. Johnson

**Affiliations:** 1 Centre for Infectious Disease Epidemiology and Research, Faculty of Health Sciences, University of Cape Town, Anzio Road, Cape Town, 7925 Observatory South Africa; 2 Institute for Global Health, UCL, London, UK; 3 Burnet Institute, Melbourne, Australia; 4 Department of Decision Sciences, University of South Africa, Pretoria, South Africa; 5 Avenir Health, Glastonbury, USA; 6 Department of Population Health, NYU Grossman School of Medicine, New York, USA; 7 Center for Global Health and Development, Boston University, Boston, USA; 8 Health Economics and Epidemiology Research Office, University of the Witwatersrand, Johannesburg, South Africa; 9 Department of Medical Microbiology, Amsterdam University Medical Centre, Amsterdam, The Netherlands

**Keywords:** HIV, Mathematical modelling, Epidemic projections, Model comparison, Coefficient of variation

## Abstract

**Background:**

Mathematical models are increasingly used to inform HIV policy and planning. Comparing estimates obtained using different mathematical models can test the robustness of estimates and highlight research gaps. As part of a larger project aiming to determine the optimal allocation of funding for HIV services, in this study we compare projections from five mathematical models of the HIV epidemic in South Africa: EMOD-HIV, Goals, HIV-Synthesis, Optima, and Thembisa.

**Methods:**

The five modelling groups produced estimates of the total population, HIV incidence, HIV prevalence, proportion of people living with HIV who are diagnosed, ART coverage, proportion of those on ART who are virally suppressed, AIDS-related deaths, total deaths, and the proportion of adult males who are circumcised. Estimates were made under a “status quo” scenario for the period 1990 to 2040. For each output variable we assessed the consistency of model estimates by calculating the coefficient of variation and examining the trend over time.

**Results:**

For most outputs there was significant inter-model variability between 1990 and 2005, when limited data was available for calibration, good consistency from 2005 to 2025, and increasing variability towards the end of the projection period. Estimates of HIV incidence, deaths in people living with HIV, and total deaths displayed the largest long-term variability, with standard deviations between 35 and 65% of the cross-model means. Despite this variability, all models predicted a gradual decline in HIV incidence in the long-term. Projections related to the UNAIDS 95–95-95 targets were more consistent, with the coefficients of variation below 0.1 for all groups except children.

**Conclusions:**

While models produced consistent estimates for several outputs, there are areas of variability that should be investigated. This is important if projections are to be used in subsequent cost-effectiveness studies.

**Supplementary Information:**

The online version contains supplementary material available at 10.1186/s12889-023-16995-9.

## Background

The UNAIDS “Fast Track” strategy to end the AIDS epidemic by 2030 aims to reduce HIV incidence and mortality by 90% over the 2010 to 2030 period [1]. Key milestones for this aim were the 90–90-90 targets for 2020 and the 95–95-95 targets for 2025 [1, 2]. South Africa, home to 20% of people living with HIV globally, did not achieve the 2020 targets, largely due to low antiretroviral treatment (ART) coverage amongst those who have been diagnosed with HIV [3, 4].

Over the years many interventions aimed at reducing the burden of HIV have been proposed and rolled out in South Africa, including condom distribution, voluntary medical male circumcision, HIV testing, and universal ART policies [5]. Decisions around how best to allocate resources across interventions are increasingly informed by mathematical models [6–8]. By projecting the course of the epidemic under different scenarios, models estimate the impact of proposed interventions, and this information allows policymakers to better determine how to achieve their targets.

However, models differ in their structures and parameterisations, and this often leads to varied projections. Inconsistencies between model outputs can suggest important research gaps. When outputs are consistent across different models despite differing underlying structures, there is typically greater confidence in their projections. A previous study comparing 12 mathematical models of the HIV epidemic in South Africa found that while the models produced consistent short-term estimates, there was significant variation in their long-term projections [9]. Despite the consistency in short-term estimates, a subsequent analysis comparing the projections to national survey data found that although many important trends were accurately predicted, there were some that most models did not capture (for example, most models underestimated HIV prevalence in adult men and overestimated ART coverage amongst men) [10]. This demonstrates that even when model projections are consistent, caution is still needed when drawing conclusions.

The HIV Modelling Consortium (http://www.hivmodeling.org) is a network of researchers that coordinates and supports HIV modelling, with the aim of informing policy decision-making around HIV programmes. The Modelling to Inform HIV Programmes in Sub-Saharan Africa (MIHPSA) collaboration is a core activity of the Consortium, and this collaboration aims to assess the optimal allocation of the HIV budget in South Africa and other sub-Saharan African countries. Previous HIV model comparison studies that have compared the cost-effectiveness of specific interventions have typically found that results differ mainly due to differences in the baseline estimates of the models, as opposed to assumptions regarding the rollout of interventions and their efficacy [8, 11, 12]. Consequently, in this study, which forms part of the first phase of the MIHPSA project, we aim to compare baseline epidemiological estimates from models of the HIV epidemic in South Africa.

Most previous model comparison studies have presented uncertainty ranges or confidence intervals around individual model estimates [10, 13, 14]. The extent to which confidence intervals overlap is a qualitative indicator of model agreement. While this is useful, it is less helpful in determining which outputs are subject to the greatest uncertainty. An alternative approach is to use a summary statistic to quantify the degree of consistency across model estimates. Several summary statistics have been used in the literature: kappa statistics have been used where outputs are binary, such as when determining if interventions are cost-effective [15]; correlation coefficients have been used when comparing only two quantitative estimates [16]; and ranges have been used where there are more than two estimates [17]. The standard deviation is another natural starting point for a summary statistic. However, with a variety of outputs, estimates will have very different means and units of measurement, making comparisons of ranges or standard deviations across output variables challenging. The coefficient of variation (the standard deviation divided by the mean) is therefore a more informative measure of model consistency that can be compared across outputs. The primary objective of this study is to quantify the consistency of estimates from HIV epidemic models included in the MIHPSA South Africa project. Our secondary objective is to determine the areas where model projections differ and to explore the reasons for these discrepancies.

## Methods

Affiliates of the HIV Modelling Consortium were invited to participate in the MIHPSA model comparison study. Modelling groups needed to be able to model the South African HIV epidemic, and to estimate specific outputs to be used in cost-effectiveness analyses planned for subsequent phases of the MIHPSA project. Based on these criteria, five modelling groups were selected. The models – EMOD-HIV, Goals, HIV-Synthesis, Optima, and Thembisa – have been described previously [8, 18–22], and key characteristics are presented in Table 1. Model estimates were produced between August and November 2021, and did not make provision for the impact of the COVID-19 pandemic due to the prevailing uncertainty. Participating groups were asked to produce forecasts in a “status quo” scenario (assuming continuation of current policy and pre-COVID-19 trends in service provision). Where possible, this included the projected rates of HIV testing, ART uptake, ART interruption, high risk sexual and injecting behaviours, and interventions such as voluntary medical male circumcision (VMMC) and condom use. Figure S9 in Additional file 1 shows the trends in select interventions, where recent trends can be seen to be relatively stable. Since the models differ in their structure it was not possible to achieve perfect alignment of the status quo scenario. For example, in the EMOD-HIV, HIV-Synthesis, and Thembisa models ART coverage is determined by the parameters governing ART uptake and treatment adherence, which remain constant after 2020, whereas in the Goals and Optima models the ART coverage proportion itself remains constant after 2020.

**Table 1:** characteristics of the 5 mathematical models of the HIV epidemic in South Africa

	**EMOD-HIV**	**HIV-Synthesis**	**Goals**	**Optima**	**Thembisa**
**Model structure**	Agent-based model	Agent-based model	Compartmental model	Compartmental model	Compartmental model
**Risk factors for HIV transmission**	Male circumcision, condom usage, partner concurrency, presence of sexually transmitted infections, and partner’s HIV stage and ART usage	Male circumcision, pre-exposure prophylaxis, number of condomless sexual partners in each time period, presence of sexually transmitted infections, and partner’s HIV viral load	Male circumcision, pre-exposure prophylaxis, condom usage, number of sexual partners per year, presence of sexually transmitted infections, types of sexual acts, and partner’s HIV stage and ART usage	Needle sharing, male circumcision, pre-exposure prophylaxis, condom usage, presence of sexually transmitted infections, types of sexual acts, post-exposure prophylaxis, and partner’s HIV stage and ART usage	Male circumcision, pre-exposure prophylaxis, condom usage, propensity for partner concurrency, types of sexual acts, and partner’s CD4 count, viral load, and use of ART
**Modes of HIV transmission**	Sexual and vertical	Sexual	Sexual, vertical, and sharing of needles	Sexual, vertical, and sharing of needles	Sexual and vertical
**Mortality while not on ART**	HIV mortality dependent on CD4 count and age at time of HIV infection	HIV mortality dependent on current CD4 count, viral load, and age	HIV mortality dependent on current CD4 count	HIV mortality dependent on current CD4 count and sex	HIV mortality dependent on current CD4 count, age and sex
**Infectiousness while on ART**	4% relative to untreated latent HIV	Effect mediated through viral load	4–8% relative to untreated latent HIV	0% relative to untreated latent HIV if virally suppressed, and 50% if not	Effect mediated through viral suppression, which depends on baseline CD4
**Mortality while on ART**	Dependent on baseline CD4 count, as well as age and sex	Dependent on current CD4 count and viral load, as well as age and ART regimen	Dependent on baseline CD4 count and time on ART, as well as age and sex	Dependent on current CD4 count, as well as sex	Dependent on baseline CD4 count and time on ART, as well as age and sex
**Calibration**	Optimisation algorithm used to maximise the likelihood of the observed data	Parameters sampled from distributions adjudged to be appropriate given South African data, with runs selected based on prevalence and number on ART	Parameters varied to fit outputs to prevalence and other data	Parameters varied to fit outputs to prevalence and other data	Posterior parameter distributions obtained through Bayesian estimation
**Notable differences affecting the modelling of the status quo scenario**	Circumcision rates adjusted downward to account for self-reporting and ‘partial’ circumcision	Accounts for viral resistance when determining rates of viral suppression. Does not explicitly model condom usage, but instead models the number of condomless partners	ART coverage, rates of viral suppression, and proportion circumcised remain constant in the status quo scenario (not governed, for example, by ART uptake and adherence)	ART coverage remains constant in the status quo scenario. All VMMC prior to 2018 was included in 2018 to capture cumulative VMMC until then, and annual VMMC data from 2019 onward were used as inputs	Circumcision rates adjusted downward to account for self-reporting and ‘partial’ circumcision

Common empirical data used for calibration and parameterisation are listed in Table 2. Particular emphasis was placed on calibration to HIV prevalence amongst males and females aged 15–49 years (in 2005, 2008, 2012, 2016, and 2017) and the total number of people on ART (in the years 2012 through 2020). Cause-of-death data are unreliably reported in South Africa [23], but adult vital registration is reasonably complete [24], so models were provided with data on total deaths. Calibration procedures varied between models, as outlined in Table 1, with some models performing calibration manually, and others using various statistical algorithms (such as likelihood maximisation and Bayesian estimation).

**Table 2:** common data for calibration and parameterisation, with ticks indicating where models used at least part of the provided data

**Variables**	**Data source**	**EMOD-HIV**	**HIV-Synthesis**	**Goals**	**Optima**	**Thembisa**
HIV prevalence in households	• South African National HIV Prevalence, Incidence, Behaviour and Communication Surveys from 2005, [27] 2008, [28] 2012, [29] and 2017 [30] • South Africa Demographic and Health Survey 2016 [31]	✓	✓	✓	✓	✓
ART data (number on ART by sex, and percentage virally suppressed)	• Johnson et al. (2017) [32] • ART programme analysis: Reviewing the ART programme from April 2004 to March 2014 (report by Department of Health) [33] • Private sector data from the Council for Medical Schemes • Public sector data from the District Health Information System	✓	✓	✓	✓	✓
Antenatal HIV prevalence	• Annual national HIV surveys from 1991–2017 and 2019 [34–60]			✓	✓	✓
Rates of male circumcision	• South African National HIV Prevalence, Incidence, Behaviour and Communication Survey 2017 [30] • WHO Global HIV/AIDS response: Epidemic update and health sector progress towards Universal Access: Progress Report 2011 [61] • National Department of Health Annual Reports from 2013–2019 [62–68]	✓	✓	✓	✓	✓
Condom usage	• South Africa Demographic and Health Surveys 1998, [69] 2003 and 2016 [31] • South African National HIV Prevalence, Behavioural Risks and Mass Media Household Survey 2002 [70] • South African National HIV Prevalence, Incidence, Behaviour and Communication Surveys from 2005, [27] 2008, [28] 2012, [29] and 2017 [30] • Myer (2010) [71]	✓		✓	✓	✓
Deaths	• Mortality and causes of death in South Africa: Findings from death notification 2018 (report by Statistics South Africa) [72]		✓			✓

Model estimates for the period 1990 to 2040 were compared for the following variables: total adult population (age ≥ 15 years), HIV incidence (per person-year) and prevalence amongst adults aged 15–49, the proportion of those with HIV who were diagnosed, ART coverage, the proportion of adults on ART who were virally suppressed using a threshold of 1000 RNA copies/ml, AIDS deaths, all-cause deaths amongst those aged 20–59, and the proportion of males aged 15–49 who were circumcised. Unless otherwise specified, outputs were compared for the total population, children (< 15 years), adult females, and adult males.

Most models produced estimates for the full period (1990 to 2040) for all variables. The time period for comparison of diagnosis and treatment outputs was limited: for the proportion of people living with HIV who were diagnosed the analysis was restricted to 2003 onward, when estimates for adults were available from all models (except Goals); and ART coverage and viral suppression proportions were only compared from 2005 onward.

Consistency in model estimates was assessed using coefficients of variation. For each variable, we calculated the coefficient of variation for each year, and assessed the trend over time. Where models reported viral suppression proportions using a threshold of 400 RNA copies/ml these were standardised to a threshold of 1000 RNA copies/ml following a previously-proposed adjustment [25], using the reverse Weibull distribution and a shape parameter of 2.07. Additionally, we compared the cross-model coefficients of variation with those of the individual models: where models reported 95% confidence intervals these were used to approximate corresponding standard deviations, which in turn were used to calculate coefficients of variation for individual models.

## Results

**Fig. 1:**
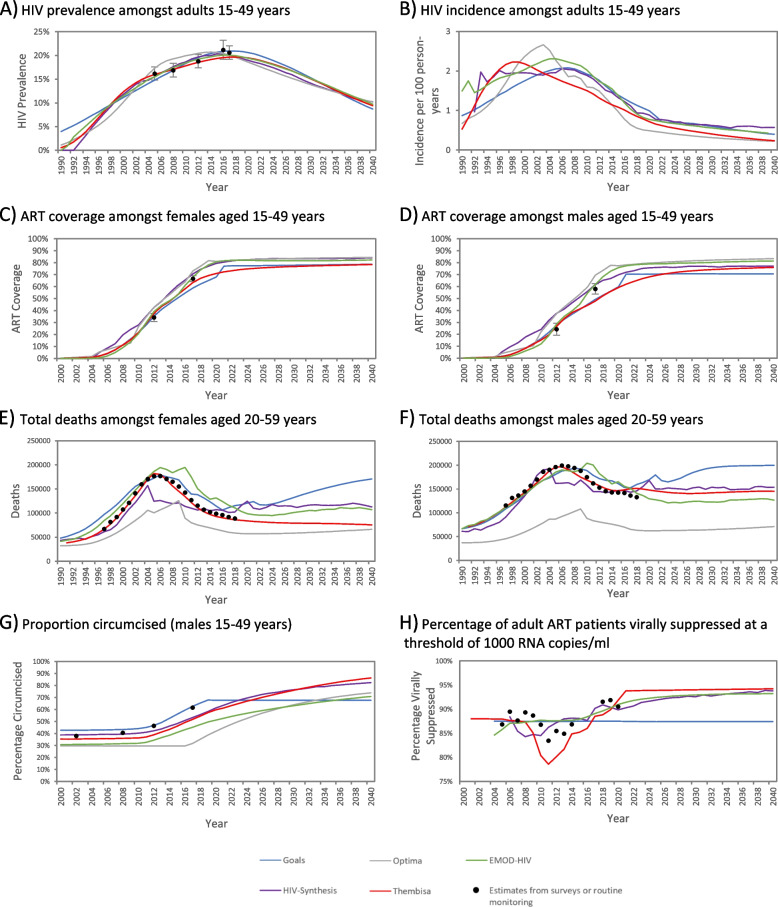
mean model outputs and survey/routine data (with 95% confidence intervals shown as vertical lines). HIV prevalence, circumcision prevalence, and ART coverage data are from the South African National HIV Prevalence, Incidence, Behaviour and Communication Surveys; death data are from Statistics South Africa (adjusted for completeness of reporting); and viral suppression data are based on the Department of Health’s ART programme monitoring

Selected outputs from the models are plotted against data in Fig. 1. Most estimates were broadly consistent with the empirical data. In certain cases, models deliberately deviated from routine/survey data. For example, in the Thembisa and EMOD-HIV models the proportion of males who were circumcised (Fig. 1G) was lower than implied by the self-reported survey data, due to concerns about the reliability of self-reporting. In other cases deviations were due to differences in model structures: the increase in the circumcision proportion was delayed in the Optima model, due to the VMMC programme only being modelled to start in 2018; and in Goals the proportion of patients who are virologically suppressed was modelled to be constant at 87.5% (Fig. 1H). An additional file presents estimates stratified by sex, as well as further outputs for children (see Additional file 1).

**Fig. 2:**
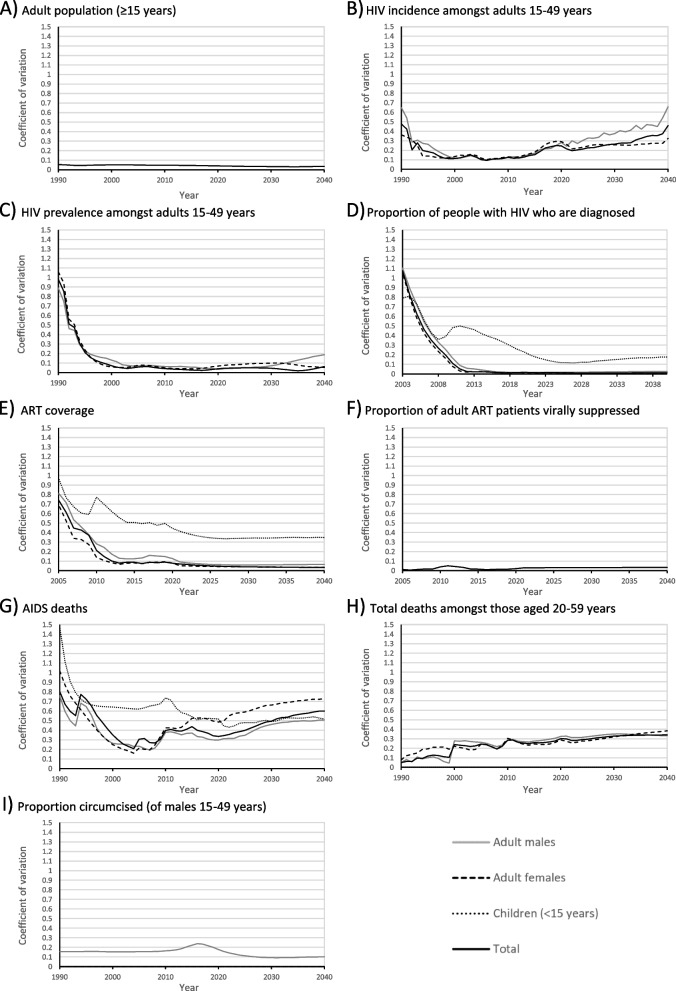
trends in the coefficients of variation

The coefficients of variation are presented in Fig. 2. For most outputs the coefficients of variation are initially relatively large due to there being limited data available for calibration in the 1990s. As more data became available from surveys and routine monitoring, most estimates became relatively consistent between 2005 and 2025, with coefficients of variation only increasing towards the end of the projection period. Estimates of the total adult population size were very consistent, with the coefficient of variation remaining below 0.05 throughout the latter half of the projection period (Fig. 2A) – i.e. the standard deviation of model estimates was less than 5% of the mean. Estimates of HIV incidence showed greater long-term variation (Fig. 2B): the standard deviation for HIV incidence in 2040 was approximately 33% and 65% of the means for females and males, respectively. Despite the variation in HIV incidence, the emphasis on calibration to prevalence data meant that estimates of HIV prevalence displayed significant consistency, with the coefficients of variation remaining below 0.1 for most of the projection period (Fig. 2C).

In terms of the 95–95-95 targets, the models produced consistent estimates (coefficient of variation below 0.1) for the proportion of adults with HIV who were diagnosed (Fig. 2D), the ART coverage in adults (Fig. 2E), and the proportion of adult ART patients who were virally suppressed (Fig. 2F), but there was higher variation in estimates for children. Apart from a brief spike from 2010 to 2013 (due to a sharp decline in the viral suppression proportion modelled by Thembisa from 2006 to 2011) the coefficient of variation for viral load suppression in adults remained below 0.04 throughout the projection period. The slow upward trend in the long-term is due to the use of a constant proportion of viral suppression in the Goals model (87.5%), while estimates from other models converged to 94%.

Estimates of AIDS deaths (Fig. 2G) and total deaths (Fig. 2H) were less consistent, and showed steady increases in variability, with long-term standard deviations of approximately 60% and 35%, respectively. For the proportion circumcised the coefficient of variation starts rising in 2010 and peaks in 2016 (Fig. 2I) – corresponding with the period during which the rates of circumcision (Fig. 1G) are rising in all models besides Optima, which only models VMMC implementation from 2018 (the version of Optima used in this study did not distinguish between VMMC and traditional circumcision in prior years). The slow increase in later years is due to the plateau in the proportion circumcised in the Goals model.

**Fig. 3:**
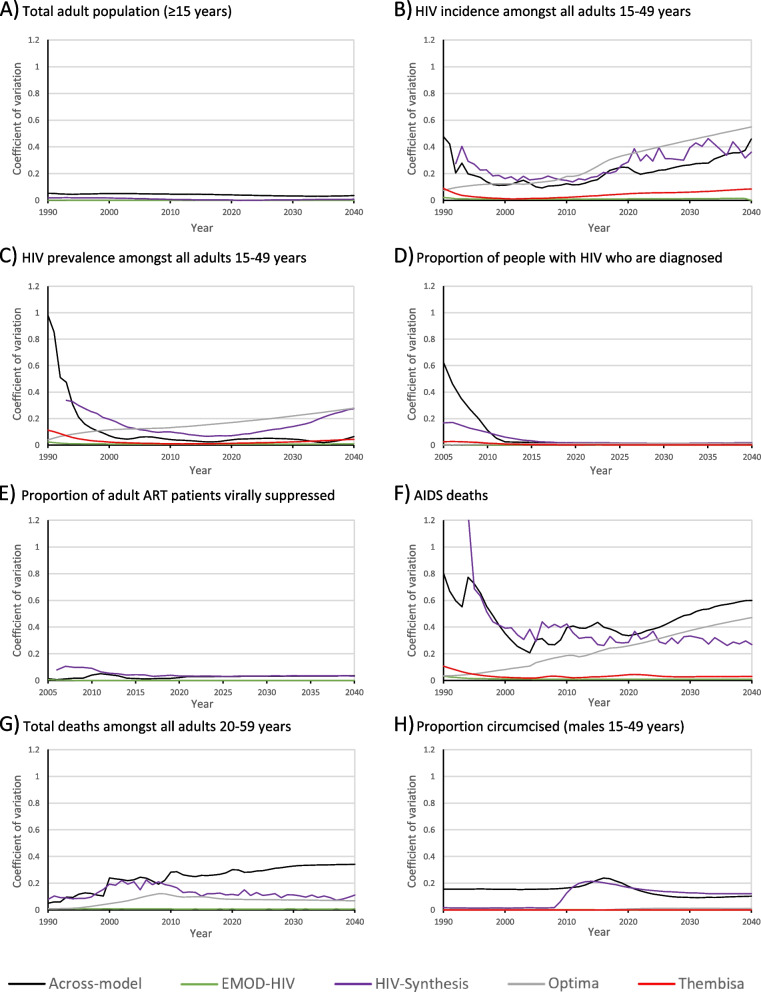
comparisons of cross-model coefficients of variation (COVs) with individual COVs calculated from 95% confidence intervals. For certain outputs confidence intervals were not obtained from all models: total adult population (**A**) and proportion virally suppressed (**E**) do not include Optima and Thembisa, and total deaths in adults 20–59 years (**G**) does not include Thembisa

The 95% confidence intervals available from individual models were generally small relative to the cross-model variability of projections (Fig. 3), although the Optima and HIV-Synthesis models had notably wider confidence intervals than the EMOD-HIV and Thembisa models (the Goals model did not produce 95% confidence intervals for this study). For the 95–95-95 programme indicators the standard deviations of model estimates were below 4% of the cross-model means, and models projected that by 2040, in a status quo scenario, approximately 96% of adults with HIV would be diagnosed, 80% of adults with HIV would be receiving ART, and 92% of adult ART patients would be virally suppressed.

## Discussion

In this study we compared the consistency of epidemiological projections from five models of the HIV epidemic in South Africa, under a status quo scenario. As measured by coefficients of variation, there was reasonable consistency between 2005 and 2025, with increasing variability toward the end of the projection period. The greatest variability was found in the projections of HIV incidence, AIDS-related deaths, and total deaths, where the standard deviations of model estimates were found to be up to 65% of the cross-model means. Despite this variability, all models encouragingly predicted a gradual decline in HIV incidence in the long-term. Model projections were notably consistent regarding the 95–95-95 targets amongst adults, and all five models predicted that the main programme ‘gap’ is poor ART coverage. However, there was more variability in estimates of the 95–95-95 targets in children.

We observed wide variation in the level of uncertainty reported by models, which likely arise from differences in the data used for calibration. For example, the HIV-Synthesis and Optima models were not calibrated to antenatal HIV prevalence data, which are a major source of information on trends in HIV prevalence. Confidence interval widths may also be a reflection of the extent to which data uncertainty is incorporated in the calibration process. For example, the Thembisa model does not consider uncertainty in male circumcision rates in model calibration, and therefore produces coefficients of variation close to zero for estimates of male circumcision prevalence. For AIDS-related deaths and total deaths we found that future projections exhibited greater variation across models than uncertainty within any given model.

Multi-model comparisons offer an important opportunity to test the robustness of model predictions to variations in model structure, assumptions, and calibration methodologies. These variations reflect different understandings and beliefs about the dynamics of the epidemic, as well as different views about acceptable trade-offs between simplicity and realism. Although these differences of opinion should be respected, these differences lead to inter-model discrepancies and, as found in this study, it is particularly important to consider which data sources were used for model calibration. With no previous studies having quantified the consistency of estimates from a set of mathematical epidemiology models, our study establishes a baseline for this quantification. Future model comparison studies may be able to judge their measures of consistency against those obtained in this study.

In prior work by the HIV Modelling Consortium to evaluate the potential impact and cost-effectiveness of “Treat All” HIV guidelines, we found that models produced results that were consistent in terms of policy implications, despite having a wide variety of model characteristics and baseline epidemic projections [26]. However, as model-based HIV analyses become more nuanced in the types of policy trade-offs being examined, differences in baseline projections are likely to have greater influence on the ultimate policy implications of model outputs. In particular, cost-effectiveness estimates have been found to be sensitive to HIV incidence and AIDS-related deaths [8, 11, 12], both of which were found to have significant cross-model variability in this study. Our study lays a foundation for understanding similarities and differences in the outcomes of future policy analyses, such as the relative cost-effectiveness and optimal budget allocation across HIV services in South Africa. Ultimately, these analyses will reflect both the differences in baseline epidemic trajectories analysed here, and differences in model assumptions regarding the costs and impacts of specific policy options. Our analysis presents a first step toward “teasing apart” potential reasons for similarities and differences in policy implications derived from HIV modelling.

This study has several limitations. First, there are other models of the HIV epidemic in South Africa that were not included in this study. This is due to the inclusion criteria requiring specific health economic outputs for a broad range of HIV interventions, planned in later phases of the MIHPSA project. Second, due to differing model structures the status quo scenario varied slightly between models. For example, some models assumed that certain variables (such as the circumcision prevalence, ART coverage, and levels of viral suppression) were constant in the status quo scenario, whereas in others these variables continued to change as they were dynamically modelled processes (albeit with time-constant inputs). Third, numerous sources of calibration data were available, but models used various subsets of these data as appropriate for their structures. While recognising the value of variation in model structures and calibration methodologies, alignment to a common set of calibration data would likely improve the consistency of model projections. Fourth, some models did not produce results for all output variables. This limits our ability to find the areas of greatest uncertainty and refine the modelling of these aspects of the epidemic.

Lastly, the coefficient of variation does have potential pitfalls. Several model outputs that are proportions, such as ART coverage and viral suppression, are projected to approach 100% in the future, leading to coefficients of variation trending to zero. Conversely, certain estimates such as HIV incidence may approach 0%, leading to very high coefficients of variation. In these cases it may be better to apply a logit transformation to highlight differences across models for such outcomes. This was attempted in the current study, but produced distortions in these indicators at earlier timepoints when their values were far below 100%. Alternative transformations of model outcomes, informed by contemporary HIV epidemic goals and benchmarks, may be required to contextualize our findings for future analyses. Future studies may also consider alternative measures of consistency, such as the intra-model coefficient of variation of an ensemble model. However, the construction of the ensemble model’s prediction intervals would need careful consideration to avoid conflating within-model uncertainty with inter-model variation.

## Conclusion

Evaluating consistencies and differences in model projections can help to set the stage for policy analyses and highlight areas for future research. Our study found consistent estimates in population sizes, HIV prevalence, and the 95–95-95 indicators in adults, but observed wider variation in paediatric 95–95-95 indicators, HIV incidence and both AIDS-related and overall deaths. Additional data collection and inclusion in model calibration procedures, particularly regarding HIV incidence and paediatric HIV service coverage, will be necessary to reduce uncertainty in HIV epidemic trends in South Africa.

### Supplementary Information

**Additional file 1: Figure S1.** AIDS deaths amongst adult males. **Figure S2.** AIDS deaths amongst adult females. **Figure S3.** HIV incidence amongst females aged 15-49 years. **Figure S4.** HIV incidence amongst males aged 15-49 years. **Figure S5.** proportion of adult females with HIV who are diagnosed. **Figure S6.** proportion of adult males with HIV who are diagnosed. **Figure S7.** proportion of children with HIV who are diagnosed. **Figure S8.** ART coverage in children. **Figure S9.** recent trends in select interventions.

## Data Availability

The data generated and analysed in this study are available from the corresponding author on request.

## Abbreviations

ART: Antiretroviral treatment
MIHPSA: Modelling to Inform HIV Programmes in Sub-Saharan Africa
VMMC: Voluntary medical male circumcision

## References

[1] UNAIDS. Fast-Track: Ending the AIDS Epidemic by 2030. Joint United Nations Programme on HIV/AIDS; 2014 . Available from: https://www.unaids.org/en/resources/documents/2014/JC2686_WAD2014report. Cited 2022 Jul 29.

[2] UNAIDS. IN DANGER: UNAIDS Global AIDS Update 2022. Joint United Nations Programme on HIV/AIDS; 2022 . Available from: https://www.unaids.org/en/resources/documents/2022/in-danger-global-aids-update. Cited 2022 Jul 28.

[3] UNAIDS. Seizing the Moment: UNAIDS Global AIDS Update 2020. Joint United Nations Programme on HIV/AIDS; 2020 . Available from: https://www.unaids.org/en/resources/documents/2020/global-aids-report. Cited 2022 Aug 24.

[4] UNAIDS. Confronting Inequalities: UNAIDS Global AIDS Update 2021. Joint United Nations Programme on HIV/AIDS; 2021 . Available from: https://www.unaids.org/en/resources/documents/2021/2021-global-aids-update. Cited 2022 Jul 28.

[5] Hopkins KL, Doherty T, Gray GE (2018). Will the current National Strategic Plan enable South Africa to end AIDS, tuberculosis and sexually transmitted infections by 2022?. South Afr J HIV Med.

[6] Stuart RM, Grobicki L, Haghparast-Bidgoli H, Panovska-Griffiths J, Skordis J, Keiser O (2018). How should HIV resources be allocated? Lessons learnt from applying Optima HIV in 23 countries. J Int AIDS Soc.

[7] Avanceña AL, Hutton DW (2020). Optimization models for HIV/AIDS resource allocation: A systematic review. Value in Health.

[8] Bansi-Matharu L, Mudimu E, Martin-Hughes R, Hamilton M, Johnson L, Ten-Brink D (2022). Cost-effectiveness of voluntary male medical circumcision (VMMC) for HIV prevention across sub-Saharan Africa: results from five independent models.

[9] Eaton JW, Johnson LF, Salomon JA, Bärnighausen T, Bendavid E, Bershteyn A (2012). HIV treatment as prevention: systematic comparison of mathematical models of the potential impact of antiretroviral therapy on HIV incidence in South Africa. PLoS Med.

[10] Eaton JW, Bacaër N, Bershteyn A, Cambiano V, Cori A, Dorrington RE (2015). Assessment of epidemic projections using recent HIV survey data in South Africa: a validation analysis of ten mathematical models of HIV epidemiology in the antiretroviral therapy era. Lancet Glob Health.

[11] Korenromp EL, Bershteyn A, Mudimu E, Weiner R, Bonecwe C, Loykissoonlal D (2021). The impact of the program for medical male circumcision on HIV in South Africa: analysis using three epidemiological models. Gates Open Res.

[12] Bershteyn A, Jamieson L, Kim HY, Platais I, Milali MP, Mudimu E (2022). Transmission reduction, health benefits, and upper-bound costs of interventions to improve retention on antiretroviral therapy: a combined analysis of three mathematical models. Lancet Glob Health.

[13] Hontelez JA, Lurie MN, Bärnighausen T, Bakker R, Baltussen R, Tanser F (2013). Elimination of HIV in South Africa through expanded access to antiretroviral therapy: a model comparison study. PLoS Med.

[14] Rehle T, Johnson L, Hallett T, Mahy M, Kim A, Odido H (2015). A comparison of South African national HIV incidence estimates: a critical appraisal of different methods. PLoS One.

[15] Jit M, Brisson M, Portnoy A, Hutubessy R (2014). Cost-effectiveness of female human papillomavirus vaccination in 179 countries: a PRIME modelling study. Lancet Glob Health.

[16] Cowie CT, Garden F, Jegasothy E, Knibbs LD, Hanigan I, Morley D (2019). Comparison of model estimates from an intra-city land use regression model with a national satellite-LUR and a regional Bayesian Maximum Entropy model, in estimating NO2 for a birth cohort in Sydney Australia. Environ Res.

[17] Brisson M, Kim JJ, Canfell K, Drolet M, Gingras G, Burger EA (2020). Impact of HPV vaccination and cervical screening on cervical cancer elimination: a comparative modelling analysis in 78 low-income and lower-middle-income countries. Lancet.

[18] Bershteyn A, Klein DJ, Wenger E, Eckhoff PA. Description of the EMOD-HIV Model v0.7 . 2012 . Available from: http://arxiv.org/abs/1206.3720. Cited 2022 Sep 5.

[19] Stover J, Hallett TB, Wu Z, Warren M, Gopalappa C, Pretorius C (2014). How Can We Get Close to Zero? The Potential Contribution of Biomedical Prevention and the Investment Framework towards an Effective Response to HIV. PLoS One.

[20] Kerr CC, Stuart RM, Gray RT, Shattock AJ, Fraser-Hurt N, Benedikt C (2015). Optima: a model for HIV epidemic analysis, program prioritization, and resource optimization. JAIDS J Acquir Immune Defic Syndr.

[21] Phillips AN, Cambiano V, Nakagawa F, Revill P, Jordan MR, Hallett TB (2018). Cost-effectiveness of public-health policy options in the presence of pretreatment NNRTI drug resistance in sub-Saharan Africa: a modelling study. Lancet HIV.

[22] Johnson L, Dorrington R. Thembisa version 4.4: A model for evaluating the impact of HIV/AIDS in South Africa. 2021 . Available from: https://www.thembisa.org/content/downloadPage/Thembisa4_4report. Cited 2022 Sep 5.

[23] Birnbaum JK, Murray CJ, Lozano R (2011). Exposing misclassified HIV/AIDS deaths in South Africa. Bull World Health Organ.

[24] Johnson LF, Dorrington RE, Laubscher R, Hoffmann CJ, Wood R, Fox MP (2015). A comparison of death recording by health centres and civil registration in South Africans receiving antiretroviral treatment. J Int AIDS Soc.

[25] Johnson LF, Kariminia A, Trickey A, Yiannoutsos CT, Ekouevi DK, Minga AK (2021). Achieving consistency in measures of HIV-1 viral suppression across countries: derivation of an adjustment based on international antiretroviral treatment cohort data. J Int AIDS Soc.

[26] Eaton JW, Menzies NA, Stover J, Cambiano V, Chindelevitch L, Cori A (2014). Health benefits, costs, and cost-effectiveness of earlier eligibility for adult antiretroviral therapy and expanded treatment coverage: a combined analysis of 12 mathematical models. Lancet Glob Health.

[27] Shisana O, Rehle T, Simbayi L. South African national HIV prevalence, HIV incidence, behaviour and communication survey, 2005. 2005 ; Available from: http://www.hsrcpress.ac.za. Cited 2005 Dec 1.

[28] Shisana O, Rehle T, Simbayi Lc, Zuma K, Jooste S, Wyk PV, et al. South African national HIV prevalence, incidence, behaviour and communication survey, 2008: a turning tide among teenagers? 2009 ; Available from: http://www.hsrcpress.ac.za. Cited 2009 Jun 9.

[29] Shisana O, Rehle T, Simbayi LC, Zuma K, Jooste S, Zungu N, et al. South African national HIV prevalence, incidence and behaviour survey, 2012. 2014 ; Available from: http://www.hsrc.ac.za/en/research-outputs/view/6871. Cited 2014 Apr 16. 10.2989/16085906.2016.1153491 27002359

[30] Simbayi L, Zuma K, Zungu N, Moyo S, Marinda E, Jooste S, et al. South African national HIV prevalence, incidence, behaviour and communication survey, 2017: towards achieving the UNAIDS 90–90–90 targets. 2019 ; Available from: https://www.hsrcpress.ac.za/books/south-african-national-hiv-prevalence-incidence-behaviour-and-communication-survey-2017. Cited 2019 Nov 6.

[31] National Department of Health (NDoH), Statistics South Africa (Stats SA), South African Medical Research Council (SAMRC), ICF. South Africa demographic and health survey 2016. Pretoria, South Africa and Rockville, Maryland, USA. 2019 ; Available from: https://www.dhsprogram.com/pubs/pdf/FR337/FR337.pdf. Cited 2019 Mar 19.

[32] Johnson LF, Dorrington RE, Moolla H (2017). Progress towards the 2020 targets for HIV diagnosis and antiretroviral treatment in South Africa. South Afr J HIV Med.

[33] Department of Health (2015). ART programme analysis: Reviewing the ART programme from April 2004 to March 2014.

[34] Kustner H (1991). First national HIV survey of women attending antenatal clinics, South Africa, October/November 1990. Epidemiol Comments.

[35] Swanevelder R (1992). Second national HIV survey of women attending antenatal clinics, South Africa, October/November 1991. Epidemiol Comments.

[36] Swanevelder R (1993). Third national HIV survey of women attending antenatal clinics, South Africa, October/November 1992. Epidemiol Comments.

[37] Kustner HGV, Swanevelder JP, Van Middelkoop A (1994). National HIV surveillance-South Africa, 1990–1992. S Afr Med J.

[38] Swanevelder R (1994). Fourth national HIV survey of women attending antenatal clinics, South Africa, October/November 1993. Epidemiol Comments.

[39] Department of Health (1995). Fifth national HIV survey in women attending antenatal clinics of the public health services in South Africa, October/November 1994. Epidemiol Comments.

[40] Department of Health. Sixth National HIV survey of women attending antenatal clinics of the public health services in the Republic of South Africa, October/November 1995. Epidemiological Comments. 2006;23(1):3–17.

[41] Department of Health (1997). Seventh national HIV survey of women attending antenatal clinics of the public health services, October/November 1996. Epidemiological Comments.

[42] South Africa Department of Health. Eighth Annual National HIV Sero-Prevalence Survey of Women Attending Antenatal Clinics in South Africa 1997. Pretoria: Health Systems Research & Epidemiology, report; 1998.

[43] South Africa Department of Health. 1998 National HIV sero-prevalence survey of women attending public antenatal clinics in South Africa. Pretoria: Health Systems Research & Epidemiology, report; 1999.

[44] South Africa Department of Health. National HIV Sero-Prevalence Survey of Women Attending Public Antenatal Clinics in South Africa, 1999. Pretoria: Health Systems Research & Epidemiology, report; 2000.

[45] South Africa Department of Health. National HIV and syphilis sero-prevalence survey of women attending public antenatal clinics in South Africa, 2000. Pretoria: Department of Health, report; 2001.

[46] South Africa Department of Health. National HIV and syphilis sero-prevalence survey in South Africa, 2001. Pretoria: Department of Health, report; 2002.

[47] Department of Health. National HIV and syphilis antenatal sero-prevalence survey in South Africa 2002. Department of Health; 2003 . Available from: http://www.doh.gov.za/docs/reports/2002/hiv-syphillis-f.html. Cited 2003 Sep 10.

[48] Department of Health. National HIV and syphilis antenatal sero-prevalence survey in South Africa, 2003. Department of Health; 2004 . Available from: http://www.doh.gov.za/docs/index.html. Cited 2006 Apr 9.

[49] Department of Health. National HIV and syphilis antenatal sero-prevalence survey in South Africa, 2004. Department of Health; 2005 . Available from: http://www.doh.gov.za/docs/reports/2004/hiv-syphilis.pdf. Cited 2005 Jul 11.

[50] Department of Health. National HIV and Syphilis Prevalence Survey, South Africa 2005 [Internet]. Directorate of Epidemiology and Surveillance; 2006 . Available from: http://www.doh.gov.za/docs/reports-f.html. Cited 2010 Aug 6.

[51] Department of Health. National HIV and Syphilis Prevalence Survey: South Africa, 2006 . Department of Health; 2007 . Available from: http://www.doh.gov.za/docs/reports-f.html. Cited 2007 Nov 5.

[52] Department of Health. The National HIV and Syphilis Prevalence Survey: South Africa, 2007. Department of Health; 2008 . Available from: http://www.doh.gov.za/docs/index.html. Cited 2008 Sep 1.

[53] Department of Health. 2008 National Antenatal Sentinel HIV and Syphilis Prevalence Survey. Department of Health; 2009 . Available from: http://www.doh.gov.za/docs/reports-f.html. Cited 2010 Jul 2.

[54] Department of Health. National Antenatal Sentinel HIV and Syphilis Prevalence Survey, 2009. Department of Health; 2010 . Available from: http://www.doh.gov.za/docs/reports-f.html. Cited 2011 Jul 13.

[55] Department of Health. The 2010 national antenatal sentinel HIV & syphilis prevalence survey in South Africa. Department of Health; 2011 . Available from: http://www.doh.gov.za/docs/reports/2011/hiv_aids_survey.pdf. Cited 2012 Dec 3.

[56] Department of Health. The 2011 National Antenatal Sentinel HIV and Syphilis Prevalence Survey in South Africa. Department of Health; 2012 . Available from: http://www.doh.gov.za/docs/reports/2012/Antenatal_Sentinel_survey_Report2012_final.pdf. Cited 2013 Mar 4.

[57] National Department of Health, South Africa. The 2012 National Antenatal Sentinel HIV and Herpes Simplex Type-2 Prevalence Survey in South Africa. National Department of Health; 2013 . Available from: http://www.health.gov.za/reports.php. Cited 2014 May 14.

[58] National Department of Health. The 2013 national antenatal sentinel HIV prevalence survey South Africa. National Department of Health; 2015 . Available from: http://www.health.gov.za/index.php/2014-03-17-09-09-38/reports/category/176-reports-2015. Cited 2016 May 20.

[59] National Department of Health. 2015 National Antenatal Sentinel HIV and Syphilis Survey Report. National Department of Health; 2017 . Available from: http://www.health.gov.za/index.php/shortcodes/2015-03-29-10-42-47/2015-04-30-08-18-10/2015-04-30-08-21-56?download=2584:2015-national-antenatal-hiv-prevalence-survey-final-23oct17. Cited 2019 Aug 1.

[60] Woldesenbet SA, Kufa T, Lombard C, Manda S, Ayalew K, Cheyip M, et al. The 2017 National Antenatal Sentinel HIV Survey, South Africa. National Department of Health. 2019 ; Available from: http://www.nicd.ac.za/wp-content/uploads/2019/07/Antenatal_survey-report_24July19.pdf. Cited 2019 Nov 29.

[61] World Health Organization. Global HIV/AIDS response: epidemic update and health sector progress towards universal access: progress report 2011. World Health Organization; 2011 . Available from: http://www.who.int/hiv/pub/progress_report2011/en/index.html. Cited 2012 Sep 28.

[62] National Department of Health. Annual Performance Plan: 2014/15 - 2016/17. National Department of Health; 2014 . Available from: http://www.health.gov.za/docs/strategic/2013/app201415.pdf. Cited 2014 Aug 20.

[63] National Department of Health. Annual Report 2013–2014. National Department of Health; 2014 . Available from: http://www.health.gov.za/annualreports.php. Cited 2015 Jan 25.

[64] National Department of Health. Annual Report 2014/15. National Department of Health; 2015 . Available from: http://www.health.gov.za/index.php/2014-03-17-09-09-38/2014-03-17-09-24-31/category/239-ar2015. Cited 2016 Jan 1.

[65] National Department of Health. Annual Report 2015/16. National Department of Health; 2016 . Available from: http://www.health.gov.za/index.php/2014-03-17-09-09-38/2014-03-17-09-24-31. Cited 2017 Feb 6.

[66] National Department of Health. Annual Report 2016/17. National Department of Health; 2017 . Available from: http://www.health.gov.za/index.php/2014-03-17-09-09-38/2014-03-17-09-24-31. Cited 2018 Apr 11.

[67] National Department of Health. Annual Report 2017/18. National Department of Health; 2018 . Available from: http://www.health.gov.za/index.php/2014-03-17-09-09-38/2014-03-17-09-24-31. Cited 2018 Nov 29.

[68] National Department of Health. Annual Report 2018/19. National Department of Health; 2019 . Available from: http://www.health.gov.za/index.php/2014-03-17-09-09-38/2014-03-17-09-24-31#. Cited 2020 Mar 16.

[69] Department of Health, South African Medical Research Council, MEASURE DHS. South Africa Demographic and Health Survey 1998: Full Report. Department of Health; 1999. Available from: http://dhsprogram.com/pubs/pdf/FR131/FR131.pdf.

[70] Human Sciences Research Council. South African National HIV Prevalence, Behavioural Risks and Mass Media Household Survey 2002. HSRC Press Cape Town; 2002 . Available from: http://www.hsrcpress.ac.za. Cited 2009 Feb 18.

[71] Myer L, Karim SA, Karim QA (2010). Barrier methods. HIV/AIDS in South Africa.

[72] Statistics South Africa (Stats SA). Mortality and causes of death in South Africa: Findings from death notification 2018. 2021 . Available from: http://www.statssa.gov.za/publications/P03093/P030932018.pdf. Cited 2021 Jul 26.

