# Supplementary material for: A quantitative assessment of the consistency of projections from five mathematical models of the HIV epidemic in South Africa: a model comparison study
Source: BMC Public Health. 2023 Oct 27;23:2119. doi: 10.1186/s12889-023-16995-9 (PMC10612295; doi:10.1186/s12889-023-16995-9)
Supplement: Supplementary file 1 — Additional file 1: Figure S1. AIDS deaths amongst adult males. Figure S2. AIDS deaths amongst adult females. Figure S3. HIV incidence amongst females aged 15-49 years. Figure S4. HIV incidence amongst males aged 15-49 years. Figure S5. proportion of adult females with HIV who are diagnosed. Figure S6. proportion of adult males with HIV who are diagnosed. Figure S7. proportion of children with HIV who are diagnosed. Figure S8. ART coverage in children. Figure S9. recent trends in select interventions. [file 12889_2023_16995_MOESM1_ESM.docx]

Projections stratified by sex, and further outputs for children

Figure S1: AIDS deaths amongst adult males


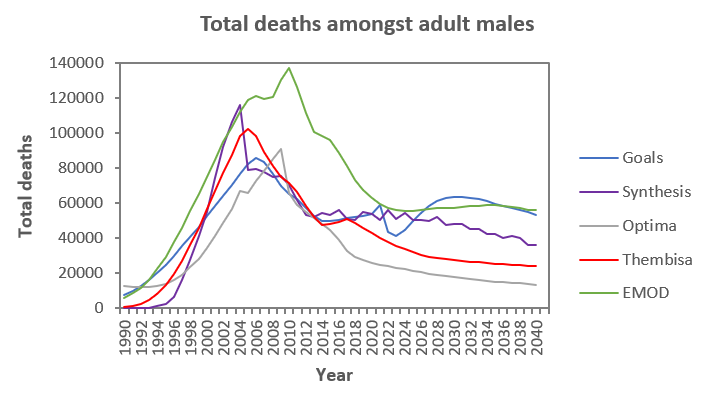


Figure S2: AIDS deaths amongst adult females


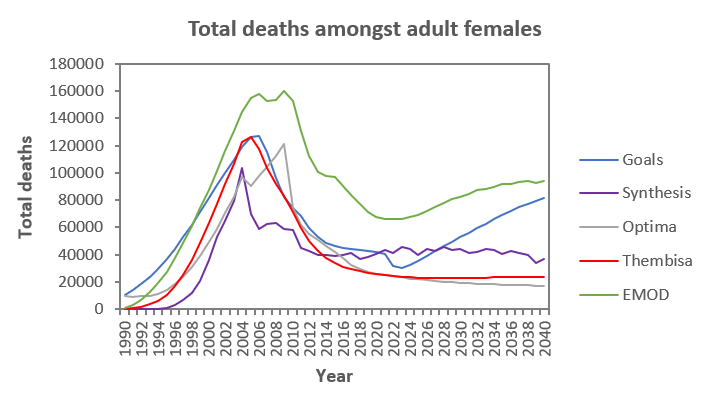


Figure S3: HIV incidence amongst females aged 15-49 years


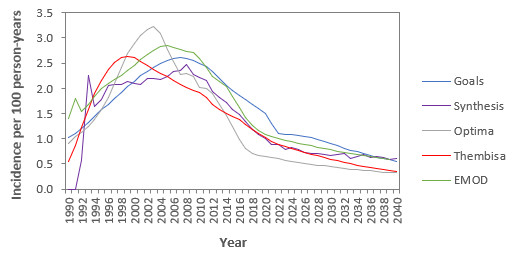


Figure S4: HIV incidence amongst males aged 15-49 years


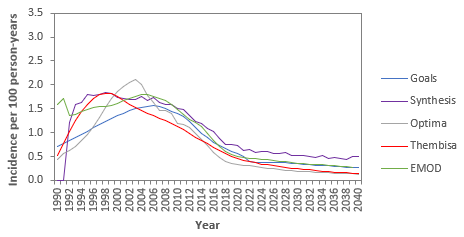


Figure S5: proportion of adult females with HIV who are diagnosed


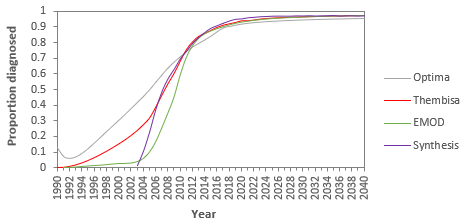


Figure S6: proportion of adult males with HIV who are diagnosed


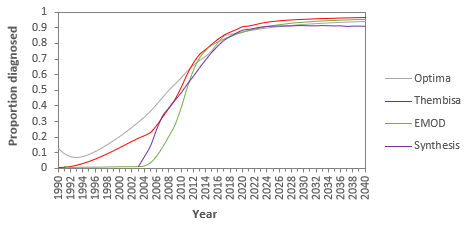


Figure S7: proportion of children with HIV who are diagnosed


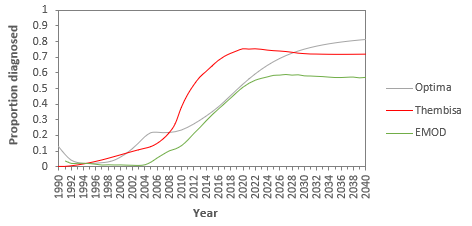


Figure S8: ART coverage in children


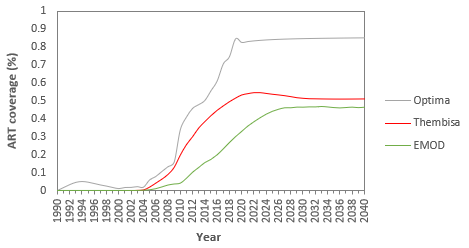


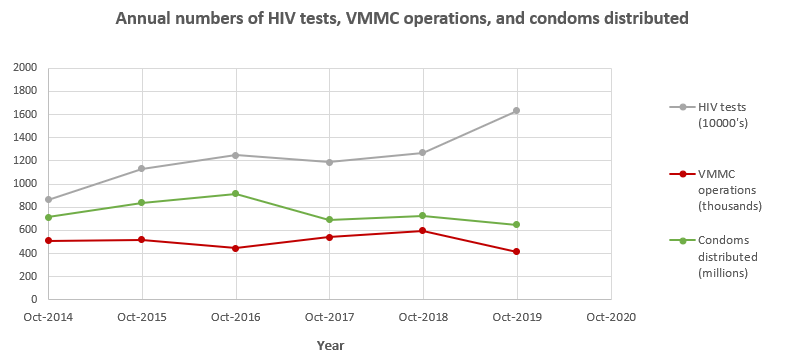
Figure S9: recent trends in select interventions

Numbers are reported for October each year to reflect the South African National Department of Health’s reporting year, which runs from April to March.
